# Optimization of Video Heart Rate Detection Based on Improved SSA Algorithm

**DOI:** 10.3390/s25020501

**Published:** 2025-01-16

**Authors:** Chengcheng Duan, Xiangyang Liang, Fei Dai

**Affiliations:** 1 School of Defence Science and Technology, Xi’an Technological University, Xi’an 710021, China; duanchengcheng@st.xatu.edu.cn; 2 School of Computer Science and Engineering, Xi’an Technological University, Xi’an 710021, China; 3 School of Sciences, Xi’an Technological University, Xi’an 710021, China; dfei@xatu.edu.cn

**Keywords:** remote photoplethysmography, heart rate, singular spectrum analysis

## Abstract

A solution to address the issues of environmental light interference in Remote Photoplethysmography (rPPG) methods is proposed in this paper. First, signals from the face’s region of interest (ROI) and background noise signals are simultaneously collected, and the two signals are processed by a differential to obtain a more accurate rPPG signal. This method effectively suppresses background noise and enhances signal quality. Secondly, the singular spectrum analysis algorithm (SSA) is enhanced to further improve the accuracy of heart rate detection. The algorithm’s parameters are adaptively optimized by integrating the spectral and periodic characteristics of the heart rate signal. Experimental results demonstrate that the method proposed in this paper effectively mitigates the effects of lighting changes on heart rate detection, thereby enhancing detection accuracy. Overall, the experiments indicate that the proposed method significantly improves the effectiveness and accuracy of heart rate detection, achieving a high level of consistency with existing contact-based detection methods.

## 1. Introduction

Heart rate (HR) is the frequency of heartbeat and is one of the most important physiological parameters in clinical diagnosis and monitoring of vital signs. The regular monitoring of heart rate is essential for good health, as it is closely associated with a variety of cardiovascular diseases (CVD), such as heart disease, arrhythmia, stroke, and hypertension [1]. CVD is a non-communicable disease, usually caused by abnormalities of the heart and blood vessels, which may lead to sudden death in severe cases. Therefore, the timely and accurate heart rate monitoring can help in the early detection of potential health risks and support effective preventive and therapeutic measures [2].

Traditional heart rate detection techniques, such as Photoplethysmography (PPG), are widely used in clinical diagnosis and health monitoring, such as finger-clip pulse oximeters and smartwatches, due to their low cost and high convenience. Thomson et al. [3] verified the heart rate detection performance of the Apple Watch and Fitbit Charge HR2 under different intensities of exercise. Paalasmaa et al. [4] used machine learning techniques to improve the accuracy of heart rate measurements; although this method is effective, the algorithm is too complex and requires high hardware. These traditional methods all rely on contact with sensors (e.g., fingers and wrists) to obtain heart rate signals, and prolonged contact may lead to discomfort, or even emotional stress or mental pressure, thus affecting the accuracy of the measurement.

With the advancement of technology, non-contact heart rate detection technology has emerged to provide an effective alternative. Devices such as microwave radar and infrared cameras have been used to achieve non-contact heart rate measurements. By irradiating an object with a wave of a fixed frequency, the object will produce a reflected wave of the same frequency; when the wave irradiates the heart, based on the Doppler effect, the frequency of the reflected wave will change, thus enabling the calculation of the heart rate. For example, Wenjie Lv et al. [5] designed a non-contact heart rate detection system using narrow-beam millimeter-wave radar to extract heart rate information by analyzing the frequency of the reflected wave, while Kaiwen Guo et al. [6] used an infrared camera to monitor the human heart rate and respiration rate. However, the high cost and poor versatility of radar and infrared cameras limit their wide application. In contrast, video-based heart rate detection methods can not only achieve the same detection accuracy as radar and infrared cameras but also have the advantages of low cost and ease of use, and thus have become a hot research topic in recent years.

**Figure 1:**
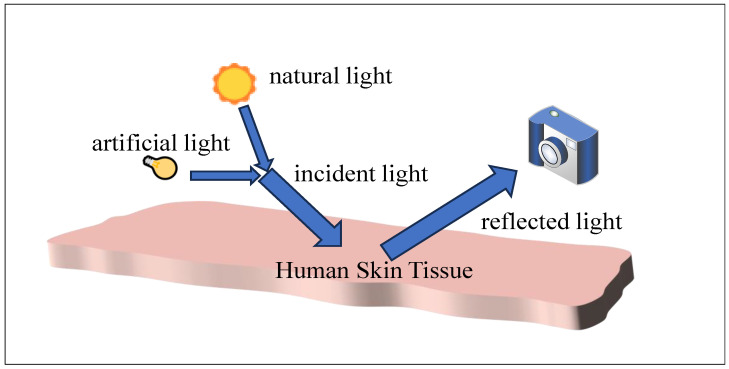
rPPG system built with sensors.

rPPG is an optical technology that measures heart rate by detecting blood changes in the face through a camera. Significant advances in rPPG technology have been made in the last decade [7,8,9]. In sensor-built rPPG systems, illumination is usually provided by a combination of natural and artificial light in the room. The illumination light hits human skin tissue and reflects the sensors, which capture a video of the human skin tissue, from which the rPPG signal is then extracted. The structure of the system is shown in Figure 1. It is based on the principle that the blood volume in the blood vessels changes periodically when the heart contracts, and this change causes corresponding periodic fluctuations in the absorption of light by the blood, which is manifested as periodic changes in the brightness of skin color. The heart rate can be deduced by recording these weak reflected light intensity changes caused by blood volume fluctuations with the sensor and analyzing them.

**Figure 2:**
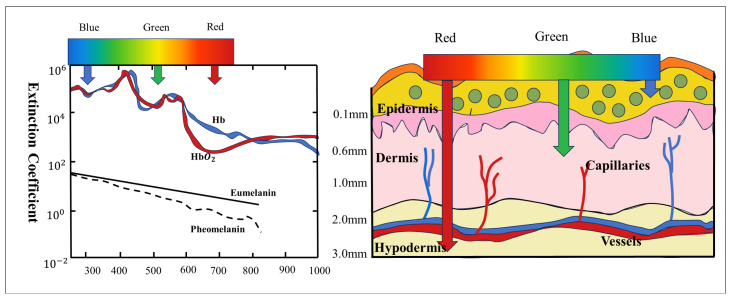
Skin optical properties of R, G, and B color channels.

The human skin has a complex tissue structure, and different substances have different absorption properties for different wavelengths of light. Figure 2 illustrates the variation of the absorbance of light by various substances with wavelength, as well as the penetration ability of light of different wavelengths into the skin. Although blood has a higher absorption near the blue spectrum (420 nm), blue light has limited ability to penetrate the skin. In contrast, deoxyhemoglobin and oxyhemoglobin have significantly higher light absorption in the green spectrum (540 nm) than red light, and the penetration ability of green light is also superior to blue light. Although the penetrating ability of red light (650 nm) is higher, its absorption by blood is lower. Therefore, in heart rate detection, the green channel of the camera has a higher signal-to-noise ratio and detection advantage compared to the red and blue channels. Based on this feature, the green channel signal is selected for acquisition and analysis in subsequent studies in this paper [10,11,12].

During rPPG signal acquisition, the facial video is affected by several factors that cause signal quality degradation. Illumination changes are one of the important interfering factors, including noise caused by changes in ambient light, such as flickering room lights, fluctuations in reflected light from computer screens, and industrial frequency noise from sensors. In addition, the subject’s movements can also affect the signal quality, including rigid movements such as head tilt and non-rigid movements such as blinking and smiling. All of these factors can cause the quality of the acquired rPPG signal to deteriorate, thus affecting the accuracy of the final heart rate detection.

In this paper, a method based on the fusion of background noise and rPPG signals is proposed to reduce the interference of sensor noise and ambient light variations on heart rate detection. The original acquired rPPG signal usually contains industrial frequency interference, ambient light variation, and motion noise. By differentiating the background noise signal from the rPPG signal, we can effectively reduce the effects of industrial frequency noise and ambient light variations on heart rate detection. In addition, we improve the SSA algorithm and use the previous heart rate detection results as feedback to further optimize the screening process of the subcomponent signals, thus improving the signal quality. The experimental results show that the proposed method can significantly reduce noise interference and significantly improve the accuracy of heart rate detection in an indoor incandescent light environment.

The remainder of the paper is organized as follows: Section 2 discusses related work, while Section 3 outlines the methodology proposed in this study. Section 4 details the experimental setting and evaluates the final results. Finally, the conclusions are drawn.

## 2. Related Work

The first step of the rPPG technique is the acquisition of signals from facial regions. The acquisition of signals from different regions can lead to large differences in the final calculation results. The main reason for this difference is that different regions of the human body do not have the same reflectance of light, and the same regions also have different reflectance values for different wavelengths of light. Currently, there are two mainstream rPPG signal extraction methods. The first method uses the whole face as the signal acquisition region, and despite its wide acquisition range, the signal noise is larger due to the sparse distribution of blood vessels in certain regions of the face. The second method, on the other hand, uses a deep learning model for face detection and selects the ROI. This method not only tracks the ROI region but also ensures accurate tracking of the target region during facial movements.

YungChien Chou et al. [13] extracted rPPG signals from the cheek and chin regions, ignoring the eye and mouth regions, which are susceptible to non-rigid motion and uneven surfaces. Yun-Yun Tsou et al. [14] deployed two 3D convolutional neural networks with shared weights to extract heart rate information from the forehead and the cheek regions, respectively, and combined the output signals of the two weighted combinations, thus effectively suppressing motion noise. Chi Zhang et al. [15] designed an rPPG-based heart rate measurement system comparing six color spaces and three color formats. By analyzing 424 captured video data, the system selected the best color channel and color space, while the best color format was determined among 10 videos. The results show that the RGB color space has the lowest mean square error in estimating heart rate and performs as the best choice of color space.

The use of an infrared camera to collect rPPG signals can effectively suppress the effects of light changes [16]. Jeanne et al. [17] used an infrared camera to estimate heart rate under conditions of ambient light changes. Although this method can effectively avoid the effect of visible light transformations on the signal, the low absorption rate of blood to infrared light compared to green light will lead to poor quality of the rPPG signal and cannot eliminate the IF interference generated by the infrared sensor. Chen et al. [18] applied the EEMD algorithm to the green channel, which is used to separate pulse wave signals from the ambient light noise; however, this method receives the cyclic light change effects. Xiaobai Li et al. [19] used the Distance Regularized Level Set Evolution (DRLSE) method to segment the background region. They used its average green value as a reference to model the illumination changes at the ROI. A normalized least mean square (NLMS) filter was used to find the optimized coefficients of the model. However, this method does not consider the industrial frequency interference of the sensor, which can lead to noise in the acquired signal.

In the field of signal processing, Richard MacEwan et al. [20] used the independent component analysis (ICA) method to analyze the RGB three-channel signals. Although this method is simpler, the robustness of the algorithm is poor, and the signal relationship between different channels is vague. Arinori Inagawa et al. [21] used principal component analysis (PCA) to process the three-channel signals, which is not only more efficient but also easier to operate than ICA. Gerard de Haan et al. [22] proposed CHROM, in which the specular reflection component is successfully eliminated by linearly combining the RGB three-channel signals, thus significantly improving the quality of the signals. Based on this, Wenjin Wang et al. [23] proposed the Plane-Orthogonal-to-Skin (POS) method, which effectively reduces the influence of skin color variations on the heart rate detection results. However, these methods all rely on RGB three-channel signals, and the signal quality of the R and B channels is usually inferior to that of the G channel. When the subject moves more, the R and B channels are prone to noise interference, which affects the signal processing and may ultimately lead to a lower signal quality than the original G channel signal. Norihiro Sugita et al. [24] on the other hand, used the SSA algorithm to process the pulse wave signal; however, they only considered the periodicity of the heart rate signal during the subcomponent screening and failed to make full use of the heart rate signal’s spectral characteristics of the heart rate signal.

## 3. Method

**Figure 3:**
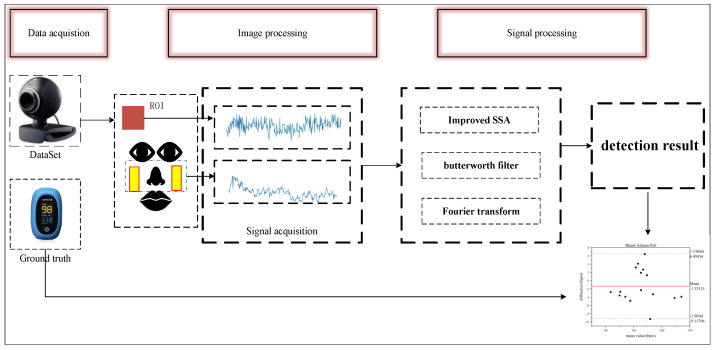
Flowchart of the method in this paper.

In this section, we provide a detailed description of the proposed heart rate detection system (see Figure 3), which is divided into two main components. First, we employ a face detection and tracking technique to identify the ROI and the background area. The average pixel values of the green channel within these two regions are calculated and utilized as signals, which are then normalized. The processed signal undergoes differential fusion to minimize noise. Subsequently, the signal is further refined using the ISSA algorithm and filtered based on the spectral characteristics of the heart rate signal. Finally, the results are transformed into the frequency domain for analysis to extract the heart rate values.

### 3.1. Extraction of the rPPG Signal

**Figure 4:**
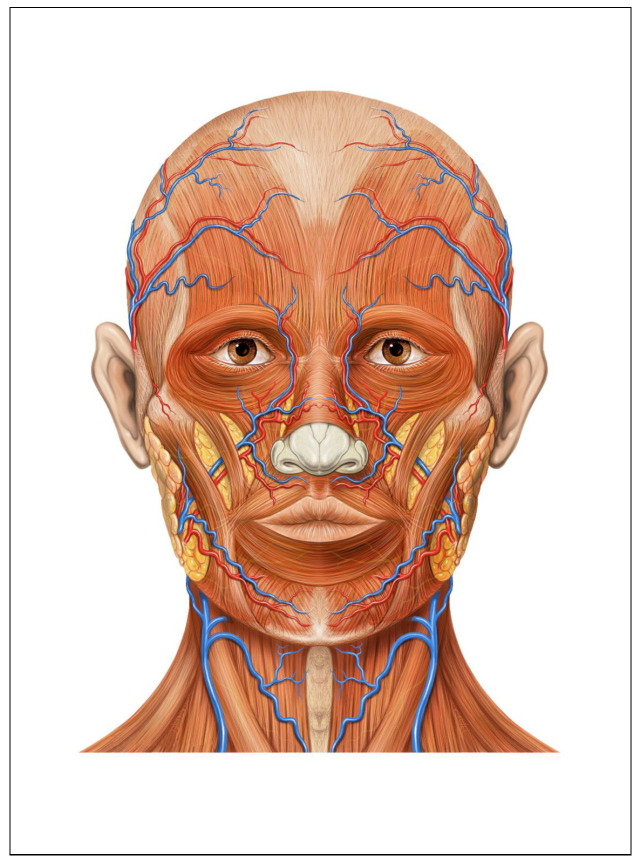
Distribution of blood vessels in the face [28].

In non-contact heart rate detection, most source signals are obtained by processing the ROI on the face. The ROIs are categorized into two main types: full-face ROIs, which are based on the face detection frame, and irregularly segmented ROIs, which are derived from facial key points, as well as localized ROIs. The full-face ROI, determined by the face detection bounding box, contains abundant facial information but also includes some background elements, which may introduce unwanted noise. If the mean value of the full-face pixels is directly used for processing, it could result in a decline in signal quality. In contrast, the irregularly segmented ROI method based on facial key points somewhat alleviates this issue. The distribution of blood vessels in the human face is illustrated in Figure 4. The forehead region is rich in blood vessels but is easily obscured by hair. Therefore, in this paper, we select the cheek region, which is abundant in blood vessels and less susceptible to non-rigid motion, for signal acquisition to enhance the accuracy and stability of the signals [25,26,27].

#### 3.1.1. Face Detection and Correction

In this paper, we utilize the MediaPipe [29] face detector, which strikes an effective balance between accuracy and detection speed when compared to face feature point detectors such as Dlib [30] and MTCNN [31]. MediaPipe can detect 468 key points on the face, which are instrumental in identifying the region of interest around the cheeks. However, despite its high detection speed and accuracy, MediaPipe may lead to the displacement of the same key points across different frames. This displacement can cause the framed region of interest to wobble due to minor facial movements, introducing noise into the acquired signal. To address this issue, we propose optimizing the MediaPipe algorithm by implementing an exponentially weighted moving average algorithm. This method mitigates the impact of single-frame detection errors on the final results by combining the detection outcomes of the latest frame with the weighted average results of historical frames. The formula for the exponentially weighted moving average algorithm is as follows:

(1) $$S_{t}=\alpha \cdot Y_{t}+\left(1-\alpha \right)S_{t-1}$$

where $S_{t}$ represents the smoothed feature point position value calculated by the algorithm, indicating the smoothing result at the current moment, $S_{t-1}$ represents the feature point position of the previous frame, $Y_{t}$ represents the feature point position of the current frame, and α is the weighting factor used to control the weighted average. In the exponentially weighted moving average (EMA) algorithm, the value of α ranges from 0 to 1. When α is closer to 1, the influence of the detection result of the current frame on the weighted average is greater, and the influence of the pixel position of the key point of the previous frame on the position of the next frame is smaller, which may lead to more obvious jitter in the region of interest; on the contrary, if the value of α is smaller, the influence of the history frame increases, and the jitter in the region of interest will be weakened, but if the value of α is taken to be too low, it may affect the accurate tracking effect of the MediaPipe algorithm on the key points. Considering these factors, this paper chooses 0.6 as the optimal value to balance the stability and tracking accuracy.

To accurately capture the rPPG signal in the ROI during facial motion, this paper introduces a facial correction technique within the facial detection process. MediaPipe can obtain the coordinates of 468 facial feature points, including the eyes, mouth, and other features. By using the center of mass coordinates of the two eyes, we can calculate the increments in the x- and y-axis directions. These increments are then utilized to determine the angle of rotation between the eyes, which is derived by applying the arctangent function (arctan). Once the rotation angle is determined, the midpoint between the two eyes serves as the center of rotation to derive the corresponding rotation matrix. Finally, the corrected facial feature point coordinates can be obtained by multiplying all 468 coordinate points by the rotation matrix. The rotation angle α from the original coordinates $\left(x,y\right)$ to the corrected coordinates $\left(x^{\prime },y^{\prime }\right)$ can be expressed using the following equation:

(2) $$\left[\begin{array}{c} x^{\prime }\\ y^{\prime } \end{array}\right]=\left[\begin{array}{cc} \cos\alpha & -\sin\alpha \\ \sin\alpha & \cos\alpha \end{array}\right]\left[\begin{array}{c} x\\ y \end{array}\right]$$

#### 3.1.2. Background Region-Based Noise Signal Cancellation

**Figure 5:**
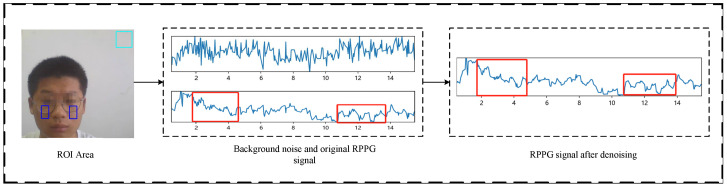
rPPG signal extraction process.

The raw rPPG signals extracted from the cheek ROI region not only contain pulse wave signals related to heart rate but also include industrial frequency interference noise generated by the camera sensor and noise caused by changes in ambient light. To effectively suppress these interference signals, a new signal processing method is proposed in this paper as shown in Figure 5. Specifically, within the background area behind the head, we frame a rectangular area of a fixed size. To reduce the influence of the subject’s head movement on heart rate detection, this rectangular region moves synchronously with the subject’s head, keeping the same angle. The signals captured in this region mainly contain noise generated by the sensor’s industrial frequency noise and ambient light variations and do not contain heart rate-related pulse wave signals. Therefore, by subtracting the signals acquired in the ROI region from the signals in this rectangular region, the pulse wave signals containing only heart rate information can be effectively extracted. The signal acquisition process can be described by the following equation:

(3) $$S\left(i\right)=\frac{\sum_{\left(x,y\right)\in ROI}R\left(x,y,i\right)}{\left|R\right|}$$

where *i* represents the *i* th frame image of the video, (x,y) represents the positional coordinates of the pixels within the ROI, R(x,y,i) represents the pixel intensities of the *i* th frame image with coordinates of (x,y), $\left|R\right|$ represents the ROI size, i.e., the total number of pixels in the ROI, and S(i) is the average pixel intensity obtained after spatial averaging.

### 3.2. Improved Singular Spectrum Analysis Algorithm

The modal decomposition of SSA is computed as follows: $X_{N}$ is the normalized rPPG signal *N* represents the signal length. The time series are systemized using a window of length *L* to form the trajectory matrix *X*. The dimensions of *X* is thus L×K, where K=N−L+1. Generally, a quarter of the signal length is used as the value for the window length *L*. In this study, *L* is set to 60. The singular value decomposition of the trajectory matrix *X* is then performed as follows:

(4) $$X=U\Sigma V^{T}$$

where Σ is the main diagonal matrix, the elements on the diagonal are the singular values, where the larger the singular value, the larger the contribution to the signal, and *u* and *V* are unit orthogonal arrays satisfying $UU^{T}=I$ and $VV^{T}=I$. We calculate the covariance matrix *S*, $S=XX^{T}$. We calculate the eigenvalues of obtaining *S*: $\lambda_{1}>\lambda_{2}>\cdots >\lambda_{L}\geq 0$, where *L* is the number of the eigenvalue, and $U_{i}$ is the eigenvector corresponding to $\lambda_{i}$. Thus, *X* can also be expressed in the form of a sum of column vectors multiplied by row vectors, which will yield *L* components:

(5) $$X=\sum_{i=1}^{L}\sqrt{\lambda_{i}}U_{i}V_{i}^{T}=X_{1}+X_{2}+\cdots +X_{L},i=1,2,\cdots ,L$$

The appropriate components are selected for recombination, each group of matrices is subjected to anti-diagonal averaging, and each group of matrices yields a sequence of length N=L+K−1. The L components are summed up to recombine the new signal sequence.

Although the background region-based noise reduction method can mitigate some noise, the acquired signals may still contain additional interfering noises, such as DC components resulting from the diastole and contraction of blood vessels, thermoregulation, or the respiration of skin tissues, as well as motion artifacts caused by the swaying of the subject’s body. These interferences diminish the signal-to-noise ratio of the signal sequence and compromise the accuracy and robustness of heart rate detection. To address these issues, this paper employs the SSA algorithm for noise reduction in the acquired signals.

The traditional SSA algorithm does not require any a priori information. It typically organizes the decomposed components based on their eigenvalues, from smallest to largest, and selects the first few components with the largest eigenvalues for reorganization. However, this original method does not consider the frequency and periodicity characteristics of heart rate signals. Therefore, in this paper, we propose a modification to the original SSA algorithm by incorporating frequency and periodicity.

In this study, the signals are decomposed using SSA, followed by applying Fast Fourier Transform (FFT) to each sub-signal and selecting the signals with a frequency range between low and high for recombination. Initially, the parameters low and high of the frequency range are set to 0.8 Hz and 3.5 Hz, respectively, aiming to cover the typical frequency band of heart rate signals. Considering that the heart rate is relatively smooth over a short period of time and does not usually vary more than 45 times per minute, we choose 0.8 as the parameter to improve the robustness of the algorithm, i.e., assuming that the heart rate varies a maximum of 48 times per minute to adjust the values of low and high. Specifically, we use the previous heart rate detection result r to dynamically adjust low and high, and the optimized parameters are low = min(low, r − 0.8) and high = max(high, r + 0.8). In this way, we reduce the filtering range and make it more adaptable to the current heart rate.

Since heart beating is a periodic physiological process, the heart rate signal also shows obvious periodicity. For this reason, in this paper, the autocorrelation function is used to determine the periodicity of the signal, whose formula is shown in Equation (6) and normalized to the interval (−1,1). When the value of the autocorrelation function of a subcomponent signal is less than 0.7, we consider that the signal is no longer periodic and eliminate it. To avoid the impact of sudden heart rate changes on the algorithm, this paper further introduces an adjustment mechanism for the difference in heart rate detection results. According to experience, if the difference between two heart rate detections is more than 30 beats per minute, we believe that the heart rate signal may lose its periodicity, so the threshold of the autocorrelation function is appropriately lowered to improve the algorithm’s ability to adapt to sudden changes in heart rate:

(6) $$R_{xx}\left[k\right]=\sum_{n=0}^{N-1}x\left[n\right]\cdot x\left[n+k\right],\;k=-\left(N-1\right),\ldots ,\left(N-1\right)$$

The pseudo-code for the improved algorithm is presented below (Algorithm 1).
**Algorithm 1:** Improved singular spectrum analysis algorithm**Input:** A segment of rPPG signal *N*, low = 0.8, high = 3.5, α=0.7, β=0.8.**While True**    $N\to X_{\left(L,K\right)}$, where K=n−L+1    $X=\sum_{i=1}^{L}\sqrt{\lambda_{i}}U_{i}V_{i}^{T}=X_{1}+X_{2}+\ldots +X_{L}$    For n=1,2,⋯,L do:         $f=\mathrm{fft}\left(X_{n}\right),\;\mathrm{acc}=\mathrm{correlate}\left(X_{n}\right)$         If low≤f≤high and acc≥α then:             $S=S+X_{n}$         End If    End For    result = fft(S)    low=min(result−β, low)    high=max(result+β, high)    If ∇result≥0.3 then:         α=0.4    End IfEnd While**Output:** Heart Rate hr=result×60

### 3.3. rPPG Signal Processing

**Figure 6:**
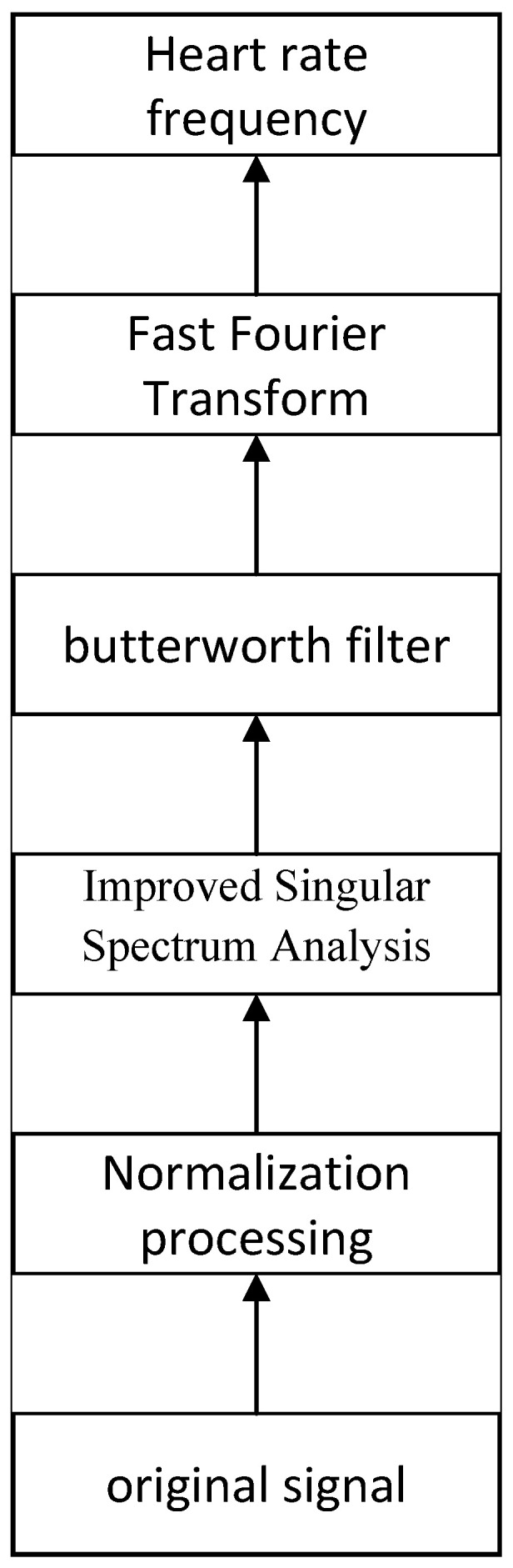
rPPG signal processing flow.

Although methods based on background noise cancellation can mitigate certain noise effects, the acquired signals may still be influenced by other sources of interference, such as DC components resulting from vasodilation and contraction, thermoregulation, or skin respiration, as well as motion artifacts caused by the subject’s body swaying. These interferences diminish the signal-to-noise ratio, thereby impacting the accuracy and reliability of physiological parameter measurements. To address these challenges, the initial rPPG signal acquired in this study is systematically processed as illustrated in Figure 6. First, the initial signals are normalized to enhance the stability and comparability of the signals across different time points and conditions, which, in turn, improves the accuracy of subsequent signal analyses. Next, the ISSA algorithm is employed to effectively eliminate noise from the signal, allowing for the extraction of the heart rate signal. Subsequently, the processed signal is filtered using a Butterworth filter to remove frequency components outside the heart rate frequency range. After filtering, the resulting pure heart rate signal is analyzed using FFT to identify the frequency with the highest power from the spectrum, enabling the calculation of the final heart rate.

**Figure 7:**
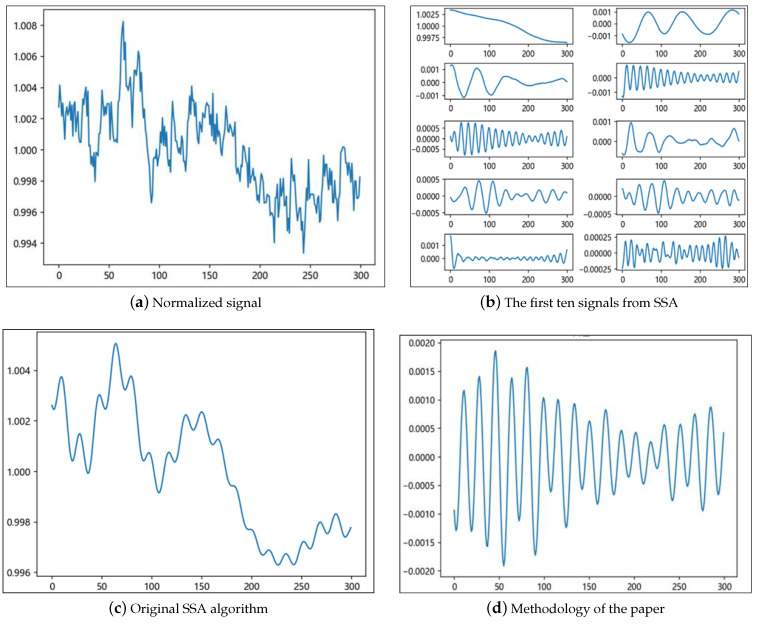
rPPG signal and signal processing for a tester.

Figure 7 illustrates the rPPG signals acquired from a tester, along with the processed signals after applying the method proposed in this paper. The original SSA algorithm utilizes the first five signals for signal reconstruction following singular value decomposition. However, as demonstrated in the comparison graph, the enhanced algorithm presented in this paper exhibits clear advantages in signal processing. This improved algorithm not only effectively eliminates baseline drift from the signal but also significantly reduces the noise component, facilitating a more accurate extraction of the pure heart rate signal.

## 4. Experimental Result and Analysis

### 4.1. Experimental Preparation

To verify the algorithm’s feasibility and accuracy, eighteen healthy volunteers aged 20 to 28 were selected for this experiment, ensuring that all participants were free from cardiovascular disease. The experiment was conducted indoors under incandescent lighting conditions, with a video capture frame rate of 30 frames per second (fps). The distance between the volunteers’ faces and the camera was approximately 0.8 m. During the experiment, the participants wore a LEPU finger-clip oximeter and remained stationary.

Three consecutive heart rate measurements were taken for each volunteer, and the average of these three measurements was recorded as a single test result. This measurement process was repeated for two rounds for each volunteer to ensure the stability and reliability of the results. Additionally, to provide accurate baseline data, heart rate results were recorded using a contact oximetry instrument, and these results were utilized as actual values for comparative analysis.

This paper compares various existing signal processing algorithms for extracting heart rate within the same experimental scenario. The algorithms studied include CHROM, POS, FastICA, EMD, and SSA. Additionally, to assess whether background denoising methods can enhance the accuracy of these algorithms, experimental comparisons of denoised and non-denoised results are conducted in this paper. The methods without background denoising are hereafter referred to as NBD, while the methods discussed in this paper are referred to as ISSA. To evaluate the performance of each algorithm, we compare their detection results with the heart rate measurements obtained through oximetry using three error metrics: mean absolute error (MAE), root mean squared error (RMSE), and Pearson correlation coefficient (ρ). These criteria quantify the discrepancy between estimated heart rate values (*Est*) and the actual values (*Rea*). The specific formulas for calculating these errors are provided below:

(7) $$M=\frac{1}{N}\sum_{i=1}^{N}\left|{Est}^{i}-{Rea}^{i}\right|$$

(8) $$R=\sqrt{\frac{1}{N}\sum_{i=1}^{N}{\left({Est}^{i}-{Rea}^{i}\right)}^{2}}$$

(9) $$\rho =\frac{cov\left(Est,Rea\right)}{\sigma \left(Est\right)\sigma \left(Rea\right)}$$

### 4.2. Experimental Result

To evaluate the performance of various heart rate extraction methods, for this paper, we conducted several heart rate experiments on 18 test subjects and process the experimental data using seven different methods, including the method proposed in this paper. The heart rate estimation results of each method are analyzed with the true heart rate values using the mean absolute error, root mean squared error, and Pearson’s correlation coefficient, and the results of their analysis are shown in Table 1. According to the experimental data, the method proposed in this paper performs well, with an average absolute error of 3.55 bpm, a root mean squared error of 3.84 bpm, and a correlation coefficient with a true heart rate value of 0.95. This method significantly outperforms the other six compared methods in all three evaluation metrics, MAE, RMSE, and ρ. These results indicate that the method of this paper outperforms the other methods in terms of accuracy and consistency, which demonstrates its validity and reliability in practical applications.

**Figure 8:**
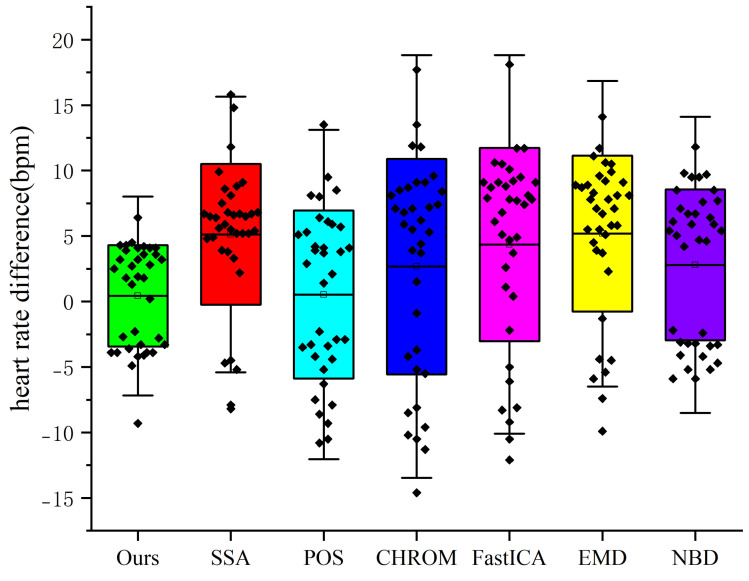
Comparison of heart rate differences using box plots.

To facilitate a more intuitive comparison of the performance of various heart rate detection methods, this paper calculates the differences between the heart rate detection results of seven methods and the true heart rate values, visualizing these differences through a box plot (see Figure 8). The results indicate that the method proposed in this paper exhibits the narrowest interquartile range of heart rate differences, centered around the zero point, with no significant outliers. This suggests that its detection results are closely aligned with the true values, demonstrating higher accuracy and stability. In contrast, the box lengths of the original SSA algorithm, the NBD method, the EMD method, and the POS method are significantly longer, suggesting that there is a large discrepancy between the results of their heart rate detections and the true values, and the MAEs of these methods are all below 7.5, while the RMSEs are in the range of 6–8. This suggests that these methods may have considerable errors in processing compared to our method, which can adversely affect the accuracy of the results. In addition, multiple outliers are evident in the box plots of CHROM and FastICA, suggesting that these methods also suffer from significant errors during heart rate detection. In conclusion, the method proposed in this paper outperforms the other methods in terms of accuracy and reliability of heart rate detection. In contrast, the other methods exhibit varying degrees of errors and outliers.

**Figure 9:**
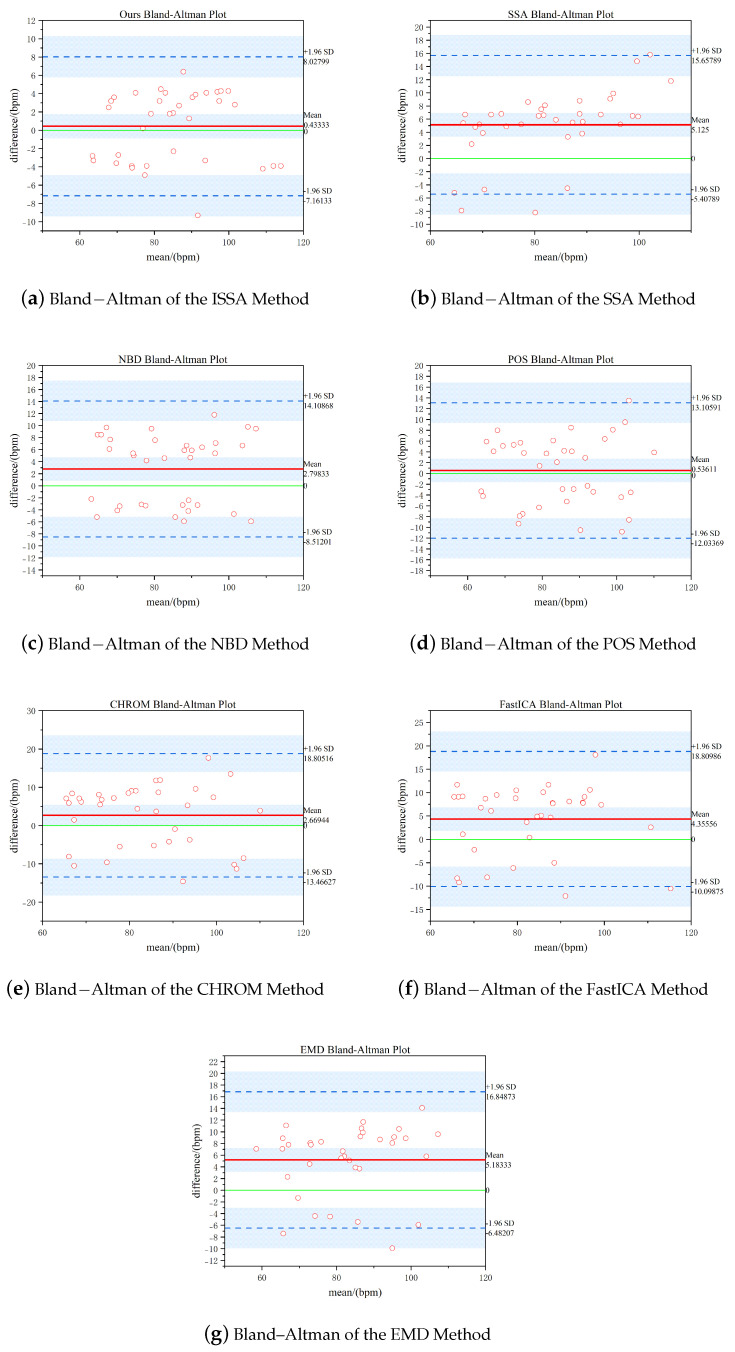
Bland-Altman consistency analysis of heart rate for the methods presented in this paper.

Bland–Altman consistency analysis is a commonly used method to assess the consistency of the results of two measurement methods. We compare the test results of oximetry with the method proposed in this paper using the Bland–Altman method, and the results are shown in Figure 9. The red solid line in the figure indicates the mean deviation of the two assay results, and the green solid line represents the 0 mean deviation. The 95% confidence interval is indicated between the two blue dashed lines. The analysis results show that the mean deviation between the oximeter and the method of this paper is 0.43 bpm, while the 95% consistency interval is [−7.16, 8.02] bpm. Almost all the results are located in the 95% confidence interval, which indicates that there is a high degree of consistency between the method of this paper and the results of the assay using the contact oximeter.

## 5. Conclusions

Aiming at the problem that the rPPG method is easily affected by the interference of ambient light and the movement of subjects in practical applications, this paper proposes a new method: the facial ROI signal and the noise signal of the background region are acquired at the same time, and the improved rPPG signals are obtained by differential processing of the two signals. This method effectively suppresses the interference of background noise and significantly improves the quality of the signal. To further improve the accuracy of heart rate detection, the SSA algorithm is improved in this paper, and the parameters of the algorithm are adaptively optimized by combining the spectral characteristics of the heart rate signal and its peripheral features. Experimental results show that the proposed method exhibits significant advantages in improving signal quality and heart rate detection accuracy.

After experimental validation, the method proposed in this paper has an average absolute error of 3.55 bpm and a root mean squared error of 3.84 bpm in heart rate detection, with a correlation coefficient of 0.95 with the results of oximetry, which shows a high consistency with the existing contact detection methods. Compared with the traditional contact measurement method, the method in this paper can be widely applied to the driver monitoring system. By installing a miniature camera device, such as a camera, on the dashboard or frame of an automobile to capture real-time video of the driver’s face and analyze his or her health status, the method can avoid direct contact with the driver, thus enhancing safety during driving. In addition, in an interrogation room, police officers can use rPPG technology to detect a suspect’s heart rate to assist in determining whether the suspect is lying, among other behaviors. These applications demonstrate the potential value of the method in a variety of real-world scenarios.

The algorithm proposed in this paper also has some limitations. The experimental subjects in this paper were mainly focused on healthy individuals between the ages of 20 and 28 years old who did not suffer from cardiovascular diseases. Therefore, future studies could further expand the age range of the subjects and include patients with cardiovascular disease to assess the applicability and reliability of the method in different populations. This will help to further expand the application of rPPG technology and promote its use in a wider range of clinical and practical scenarios.

## Figures and Tables

**Table 1:** Comparison of experimental results between the method presented in this paper and other methods.

Methods	M/(b/m)	R/(b/m)	ρ
Ours	3.55	3.84	0.95
SSA	6.81	7.37	0.92
NBD	5.9	6.34	0.9
POS	5.7	6.34	0.88
CHROM	7.79	8.54	0.82
FastICA	7.72	8.47	0.84
EMD	7.33	7.82	0.89

## Data Availability

Raw data underlying the results presented in this paper are available from the authors upon reasonable request.

## Abbreviations

The following abbreviations are used in this manuscript:

rPPG	Remote Photoplethysmography
HR	Heart Rate
SSA	Singular Spectrum Analysis
ISSA	Improved Singular Spectrum Analysis
NBD	No Background Denoising
POS	Plane Orthogonal to Skin
CHROM	Chrominance Based
FastICA	Fast Independent Component Analysis
EMD	Empirical Mode Decomposition
ROI	Region of Interest
